# Pre-pregnancy body mass index and offspring neurobehavioral development: a birth cohort study

**DOI:** 10.3389/fnut.2025.1656064

**Published:** 2025-09-25

**Authors:** Peng Sun, Youdan Hu, Min Wei, Xiuxiu Li, Zhiling Wu, Xuemei Liu, Zhaoyi Guo, Wei Li, Wei-Qing Chen, Shuya Zhong

**Affiliations:** ^1^Maternity and Child Healthcare Hospital of Nanshan District, Shenzhen, China; ^2^School of Public Health, Sun Yat-sen University, Guangzhou, China; ^3^Maternity and Child Healthcare Hospital of Nanshan District, Shenzhen, China

**Keywords:** pre-pregnancy BMI, neurobehavioral development, birth cohort, sex differences, risk factors

## Abstract

**Background:**

Neurodevelopmental disorders in children, due to their high prevalence and potential long-term adverse outcomes, require early identification and intervention. Prenatal environmental factors may affect offspring neurodevelopmental trajectories, but supporting evidence is limited. This study aims to examine the longitudinal association between maternal pre-pregnancy BMI and offspring neurobehavioral development.

**Methods:**

Based on the Shenzhen Birth Cohort established in 2018, 2,255 mother–child pairs were included. Maternal sociodemographic characteristics and pre-pregnancy BMI were obtained through prenatal questionnaires. Child neurobehavioral development from 1 to 36 months of age was assessed using the Ages and Stages Questionnaires, Third Edition (ASQ-3). Generalized estimating equations were used to analyze the association between pre-pregnancy BMI and offspring neurobehavioral development.

**Results:**

Pre-pregnancy BMI was categorized into underweight (16.1%), normal (74.9%), and overweight/obesity (9.0%). After adjusting for confounders, maternal overweight/obesity significantly increased the risk of developmental delays in the communication (aOR = 1.55, 95% CI: 1.25–1.92) and problem-solving domains (aOR = 1.44, 95% CI: 1.14–1.83). Sex-stratified analysis showed that this association was significant only in boys for the problem-solving (aOR = 1.60, 95% CI: 1.20–2.13) and personal-social domains (aOR = 1.43, 95% CI: 1.10–1.86) (*p* < 0.01).

**Conclusion:**

Maternal pre-pregnancy overweight/obesity was an independent risk factor for offspring neurobehavioral developmental delays, with sex-specific effects. These findings suggested that pre-pregnancy weight management should be included in primary prevention strategies for neurodevelopmental disorders.

## 1 Introduction

Neurodevelopmental disorders are a group of behavioral and cognitive impairments that emerge early in life, resulting from disruptions in early brain development. These disorders lead to alterations or delays in multiple developmental domains in children, including social skills and communication, language, intelligence, motor skills, attention and memory, as well as significant difficulties in acquiring and performing social functions (1). Current neurodevelopmental disorders have emerged as a global health burden, affecting over 10% of children worldwide (2, 3), and imposing substantial challenges on individuals, families, and society (4). Previous studies have shown that fewer than 30% of children with developmental delays are identified, which may delay their access to early intervention services (5, 6). Delays or alterations in specific domains of neurobehavioral development in children may affect future learning and health, with potential impacts extending into adulthood. Therefore, early screening and diagnosis are essential to enable timely intervention and support, which are critical for improving the prognosis of children with neurodevelopmental disorders (7).

Early neurodevelopmental trajectories in children are influenced by the prenatal environmental conditions (8). Prenatal exposure to adverse environmental conditions, such as maternal overweight or obesity, stress, depression or anxiety, pregnancy complications, toxins, and nutritional factors, has been associated with neurodevelopmental outcomes in children (9–11). Previous studies have suggested that maternal preconception weight may contribute to neurodevelopmental disorders in offspring by altering the developmental trajectory of the fetal nervous system. A prospective study of 1,126 mother–child pairs reported that maternal pre-pregnancy overweight/obesity was significantly associated with poorer performance across multiple domains of executive function in early childhood (e.g., inhibitory control, working memory). Maternal mid-gestation levels of high-sensitivity C-reactive protein (hs-CRP) mediated approximately 17.4% of this association, suggesting that inflammatory pathways may play an important mechanistic role. In addition, a study based on the FinnBrain Birth Cohort (*n* = 122) found that higher maternal pre-pregnancy BMI was significantly associated with increased mean diffusivity (MD) of the infant hippocampus, underscoring the importance of maternal BMI in shaping early neurodevelopmental outcomes in offspring (12, 13). Children born to women with chronic obesity, as indicated by a high pre-pregnancy body mass index (BMI), are at increased risk of suspected gross motor developmental delays. These children are also more likely to be classified as vulnerable in five major developmental domains: gross motor, fine motor, communication, problem solving and personal-social skills (14). In some countries, more than one-third of women of reproductive age are overweight or obese (15). A meta-analysis encompassing 42 epidemiological studies with 3,680,937 mother–child pairs demonstrated that maternal obesity (overweight or obese status) is associated with adverse perinatal outcomes. The study further revealed that offspring of mothers with pre-pregnancy obesity have an increased risk of developing attention-deficit/hyperactivity disorder (ADHD), autism spectrum disorder (ASD), conduct disorder, and schizophrenia. Similar elevated risks were also observed among offspring of mothers who were overweight prior to pregnancy as well as those exposed to maternal adiposity during gestation (16). However, the association between pre-pregnancy BMI and offspring neurobehavioral development remains unclear. Most existing studies have focused on a single time point of neurobehavioral assessment (17), and there is a lack of longitudinal research investigating the relationship between maternal exposure and children’s neurobehavioral development across multiple time points.

Therefore, this study aims to investigate the longitudinal association between maternal pre-pregnancy BMI and offspring neurobehavioral development outcomes through a prospective cohort study using generalized estimating equations (GEE) analysis. The primary objectives are to enhance health literacy among women of reproductive age, optimize weight management during the perinatal period, reduce the risk of neurodevelopmental impairments in offspring, and provide a scientific basis for maternal and child health interventions.

## 2 Materials and methods

### 2.1 Subjects

This study utilized data from the Shenzhen Birth Cohort (Xinmiao Project), which began recruitment in 2018 among pregnant women registered at the Maternity and Child Healthcare Hospital of Nanshan District. The cohort followed participants from the fetal stage through to the child’s seventh year of life. Data collection included questionnaire surveys and biological sample collection, aiming to establish a comprehensive mother–child health database and biobank.

The inclusion criteria for study participants comprised: (1) gestational age at registration less than 20 weeks; (2) registration at the Maternity and Child Healthcare Hospital of Nanshan District with planned prenatal care and delivery at the same hospital; (3) complete data on maternal pre-pregnancy height, weight, and delivery weight records; (4) completion of at least two ASQ-3 assessments by the mother during the 1–36 months follow-up period of the child. The exclusion criteria were as follows: (1) fetal demise, miscarriage, or multiple pregnancies during the follow-up period; (2) offspring diagnosed with major psychiatric disorders or severe intellectual disabilities; (3) incomplete data.

### 2.2 Measurement of pre-pregnancy BMI

Maternal height was measured by nurses at the time of the initial prenatal visit and recorded to the nearest 0.01 meters. Pre-pregnancy weight was self-reported by the pregnant women and documented by nurses in the maternal health handbook and electronic medical records. The classification of pre-pregnancy BMI was based on the Preconception and Pregnancy Care Guidelines (2018), published in the *Chinese Journal of Obstetrics and Gynecology* (18). This is consistent with the BMI classification standards issued by the World Health Organization (WHO) (19). According to these guidelines, underweight was defined as a BMI < 18.5 kg/m^2^, normal weight as 18.5 kg/m^2^ ≤ BMI < 25 kg/m^2^, overweight as 25 kg/m^2^ ≤ BMI < 30 kg/m^2^, and obesity as BMI ≥ 30 kg/m^2^. Since the number of cases with pre-pregnancy obesity was relatively small in this study, it was combined with the overweight group during exploratory analyses.

### 2.3 Neurobehavioral developmental assessment of offspring

This study was a hospital-based birth cohort study. The neurobehavioral development of infants and young children was assessed using the Chinese version of the *Ages & Stages Questionnaires®, Third Edition* (ASQ-3). To balance the scientific rigor of the assessment and the feasibility of follow-up, all time points for ASQ assessments were strictly set in accordance with the routine health check-up protocol for child healthcare in China. Specifically, the corresponding developmental assessments were conducted when the infants and young children were at 1, 3, 6, 8, 12, 18, 24, and 36 months of age. The ASQ-3 was designed to evaluate neurocognitive and psychomotor developmental milestones and to identify neurodevelopmental delays in children from 1 to 66 months of age. It was currently one of the most widely used screening tools for developmental delay worldwide, with good applicability across different countries (20), and demonstrated excellent test–retest reliability, internal consistency, and construct validity (21). The ASQ-3 questionnaire consisted of five sections, each containing five to six items, and was designed to assess neurobehavioral development across five key domains: communication, gross motor skills, fine motor skills, problem-solving, and personal-social skills. Based on comparisons between the obtained scores and the corresponding age-specific cutoff values, children were categorized as having normal development (scores above the cutoff) or developmental delay (scores falling within the monitoring zone or below the monitoring zone).

### 2.4 Confounding factor

The following sociodemographic characteristics were obtained through birth cohort follow-up questionnaires: (1) maternal age (≤24 years, 25–29 years, ≥30 years); paternal age (≤29 years, 30–35 years, ≥36 years); maternal ethnicity (Han, non-Han); household registration type (registered resident of Shenzhen, temporary resident, migrant); marital status (married, unmarried, other); and parental educational level (junior high school or below, senior high school, technical secondary school, junior college, university, master’s degree or above). (2) Obstetric information, including parity (0, ≥ 1), gestational hypertension (yes, no), and gestational diabetes mellitus (yes, no). Information on breastfeeding at 6 months of age (yes, no) was obtained from the child follow-up questionnaire.

The data were obtained from the Maternity and Child Healthcare Hospital of Nanshan District medical record system: (1) neonatal information, including sex (male, female), preterm birth status (yes, no), and birth weight (low birth weight, normal, macrosomia); and (2) maternal delivery information, including mode of delivery (cesarean section, vaginal delivery), and pre-delivery weight (kg). Preterm birth was defined as gestational age <37 weeks, consistent with WHO criteria.

### 2.5 Statistical analysis

Statistical analyses were performed using SPSS software (version 27.0). Two-tailed tests were applied, and a *p*-value of < 0.05 was considered statistically significant. For comparisons of continuous variables, the *t*-test or analysis of variance (ANOVA) was used if the data followed a normal distribution; otherwise, non-parametric tests were applied. The chi-square (*χ*^2^) test was used to assess differences in categorical variables, particularly to compare baseline characteristics (categorical variables) of mothers and offspring across different pre-pregnancy BMI groups. Due to the limited number of cases with pre-pregnancy obesity in this study, it was merged with the overweight group during exploratory analyses.

The data in this study involve repeated measurements. Therefore, maternal pre-pregnancy BMI was used as the independent variable, and developmental delay based on ASQ outcomes was used as the dependent variable. GEE model was applied to assess the overall effect on neurodevelopment across different ages. An exchangeable or first-order autoregressive working correlation matrix was considered, with the logit function used as the link function. The longitudinal association between maternal pre-pregnancy BMI and offspring neurobehavioral development across different functional domains was analyzed, with further sex-stratified analyses. Two models were constructed for each analysis: Model I was a crude regression model without adjustment for confounding factors, and Model II was an adjusted model controlling for multiple potential confounders. GEE is a regression model used to analyze correlated data, particularly within the framework of generalized linear models and repeated measurements. It estimates parameters using the quasi-likelihood method and can be applied to various distributions, including normal, binomial, and Poisson distributions. In this study, each child underwent a different number of ASQ developmental assessments between 1 and 36 months of age, with unequal time intervals between assessments. Moreover, repeated observations for the same child are likely to be correlated, resulting in unbalanced longitudinal data. Traditional statistical methods, such as analysis of variance, assume independence of the dependent variable and are therefore unsuitable in this context. The use of GEE allows for effective control of center effects, missing values, repeated measurement factors, and other influencing variables. It also addresses issues such as inflated test bias and invalid parameter estimates that may arise from ignoring intra-subject correlations, ultimately enabling the estimation of associated center effect values.

## 3 Results

### 3.1 Clinical baseline characteristics of the study population

A total of 2,255 mother-infant pairs were enrolled in this study. The majority of mothers were registered residents of Shenzhen (63.6%), and 50.6% were aged 30 years or older. Among fathers, the largest age group was 30–35 years, accounting for 47.4%. Both parents had relatively high educational levels, with 61.0% of mothers and 63.3% of fathers having a bachelor’s degree or higher. Additionally, 58.0% of mothers were not in their first pregnancy. Gestational diabetes was present in 14.6% of mothers, while only 1.1% had gestational hypertension. Among the offspring, 52.9% were male, and 69.9% were delivered via vaginal birth. The preterm birth rate was 4.4%, while the low birth weight rate was 3.8%. The proportions of mothers who were underweight, normal weight, overweight and obesity before pregnancy were 16.1, 74.9, and 9.0%, respectively. During pregnancy, 28.4% of mothers had inadequate gestational weight gain, 44.4% had appropriate weight gain, and 27.2% had excessive weight gain.

### 3.2 Neurobehavioral development of children

As shown in Table 1, among the five developmental domains, the rates of developmental delay were relatively higher in the communication and personal-social domains. Notably, the highest proportions of delay in both domains were observed at 1 month of age, at 36.22 and 32.46%, respectively. In terms of age distribution, the gross motor domain showed relatively better developmental outcomes across all age groups, with the lowest observed rate of developmental delay at 18 months, recorded at 1.5%.

**Table 1:** Neurobehavioral development in children at various month intervals.

Months of age (total number)	Number of children with developmental delay (*N*, %)				
	Communication	Gross motor	Fine motor	Problem solving	Personal-social
1 month (*N* = 1,027)	376 (36.6)	41 (4.0)	204 (19.9)	177 (17.2)	334 (32.5)
3 months (*N* = 1940)	342 (17.6)	245 (12.6)	437 (22.5)	388 (20.0)	490 (25.3)
6 months (*N* = 1968)	133 (6.8)	82 (4.2)	128 (6.5)	50 (2.5)	104 (5.3)
8 months (*N* = 1814)	175 (9.6)	75 (4.1)	135 (7.4)	51 (2.8)	127 (7.0)
12 months (*N* = 1806)	120 (6.6)	60 (3.3)	38 (2.1)	58 (3.2)	127 (7.0)
18 months (*N* = 1,485)	111 (7.5)	22 (1.5)	62 (4.2)	84 (5.7)	79 (5.3)
24 months (*N* = 1,456)	158 (10.9)	44 (3.0)	129 (8.9)	96 (6.6)	171 (11.7)
36 months (*N* = 1,184)	56 (4.7)	52 (4.4)	133 (11.2)	143 (12.1)	125 (10.6)

### 3.3 Association between pre-pregnancy BMI and offspring neurobehavioral development

The GEE models were constructed using maternal pre-pregnancy BMI categories as the independent variable and developmental status of the five ASQ-3 domains (normal development = 0, developmental delay = 1) across 1–36 months of age as the dependent variables (Table 2). In the unadjusted Model I, maternal pre-pregnancy overweight/obesity was associated with an increased risk of developmental delay in the communication domain (OR = 1.59, 95% CI: 1.29–1.96) and problem-solving domain (OR = 1.47, 95% CI: 1.16–1.86). Additionally, maternal pre-pregnancy underweight was identified as a risk factor for developmental delay in the problem-solving domain (OR = 1.23, 95% CI: 1.01–1.50). After adjustment for covariates in Model II, compared to mothers with normal pre-pregnancy BMI, pre-pregnancy overweight/obesity remained associated with an increased risk of developmental delay in the communication domain (OR = 1.55, 95% CI: 1.25–1.92) and problem-solving domain (OR = 1.44, 95% CI: 1.14–1.83) of offspring. In addition, after adjustment in Model II, maternal pre-pregnancy underweight was no longer a significant risk factor for developmental delay in the problem-solving domain.

**Table 2:** The association between maternal pre-pregnancy BMI and offspring neurobehavioral development.

Developmental domains	Pre-pregnancy BMI	Model I	*P*	Model II	*P*
		OR (95% CI)		aOR (95% CI)	
Communication					
	Normal BMI	1.00		1.00	
	Underweight	1.14 (0.95, 1.36)	0.15	1.09 (0.92, 1.31)	0.32
	Overweight/obesity	1.59 (1.30, 1.96)	<0.001	1.55 (1.25, 1.92)	<0.001
Gross motor					
	Normal BMI	1.00		1.00	
	Underweight	1.18 (0.92, 1.52)	0.20	1.1 (0.85, 1.41)	0.48
	Overweight/obesity	1.15 (0.82, 1.63)	0.42	1.18 (0.83, 1.68)	0.35
Fine motor					
	Normal BMI	1.00		1.00	
	Underweight	1.11 (0.92,1.34)	0.28	1.06 (0.88, 1.28)	0.55
	Overweight/obesity	1.06 (0.84,1.34)	0.65	1.04 (0.82, 1.32)	0.76
Problem solving					
	Normal BMI	1.00		1.00	
	Underweight	1.23 (1.01, 1.50)	0.04	1.19 (0.98, 1.46)	0.08
	Overweight/obesity	1.47 (1.16, 1.86)	<0.001	1.44 (1.14, 1.83)	<0.001
Personal-social					
	Normal BMI	1.00		1.00	
	Underweight	1.11 (0.92, 1.32)	0.27	1.04 (0.87, 1.24)	0.69
	Overweight/obesity	1.19 (0.95, 1.48)	0.13	1.2 (0.96, 1.5)	0.10

Model I: unadjusted for confounding factors; Model II: adjusted for confounding factors, including maternal age, paternal age, household registration location, gestational hypertension, gestational diabetes, parity, gestational weight gain, child sex, preterm birth, birth weight, and breastfeeding status at 6 months of age.

Further subgroup analysis by child sex showed that (Table 3), compared to mothers with normal pre-pregnancy BMI, pre-pregnancy overweight/obesity was a significant risk factor for developmental delay in the communication domain, in both girls (1.39, 95% CI: 1.01–1.93) and boys (1.62, 95% CI: 1.24–2.13), both reaching statistical significance. However, in the problem-solving and personal-social domains, the adverse effects of pre-pregnancy overweight/obesity on developmental delay were significant only in boys. The odds ratios (95% CI) were 1.60 (1.20–2.13) for the problem-solving domain and 1.43 (1.10–1.86) for the personal-social domain.

**Table 3:** Child sex-specific associations between maternal pre-pregnancy BMI and offspring neurobehavioral development.

Developmental domains	Pre-pregnancy BMI	Girls		Boys	
		aOR (95% CI)	*P*	aOR (95% CI)	*P*
Communication					
	Normal BMI	1.00		1.00	
	Underweight	1.13 (0.86, 1.49)	0.38	1.09 (0.86, 1.36)	0.48
	Overweight/obesity	1.39 (1.01, 1.93)	0.046	1.62 (1.24, 2.13)	<0.001
Gross motor					
	Normal BMI	1.00		1.00	
	Underweight	1.02 (0.69, 1.50)	0.92	1.25 (0.90, 1.74)	0.19
	Overweight/obesity	0.73 (0.43, 1.26)	0.26	1.48 (0.94, 2.34)	0.09
Fine motor					
	Normal BMI	1.00		1.00	
	Underweight	0.99 (0.76, 1.3)	0.96	1.13 (0.87, 1.47)	0.36
	Overweight/obesity	0.85 (0.59, 1.21)	0.36	1.20 (0.89, 1.63)	0.24
Problem solving					
	Normal BMI	1.00		1.00	
	Underweight	1.27 (0.94, 1.71)	0.12	1.18 (0.91, 1.54)	0.21
	Overweight/obesity	1.21 (0.81, 1.8)	0.35	1.60 (1.20, 2.13)	0.002
Personal-social					
	Normal BMI	1.00		1.00	
	Underweight	1.06 (0.81, 1.39)	0.66	1.06 (0.83, 1.35)	0.66
	Overweight/obesity	0.79 (0.52, 1.20)	0.28	1.43 (1.10, 1.86)	0.007

## 4 Discussion

Among the 2,255 mothers included in this study, 363 (16.1%) were underweight before pregnancy, 191 (8.5%) were overweight, and only 12 (0.5%) were classified as obese prior to pregnancy. According to a 2021 nationwide survey on the prevalence of overweight and obesity among adults across nine provinces in China (22), 33.3% of women were overweight and 14.6% were obese. These data were notably higher than the proportions of pre-pregnancy overweight and obesity observed in the mothers from this study. This discrepancy may be attributed to the increase in body fat percentage with age among adult women, as well as variations in obesity rates across different age groups. In addition, over 60% of the participants in our study were registered residents of Shenzhen, a highly developed urban area, and both parents had relatively high educational levels. These factors may contribute to greater attention to body weight and dietary management. Therefore, the differences from the national survey may also reflect regional variations in social and dietary habits.

Our findings suggested that maternal pre-pregnancy overweight/obesity increased the risk of developmental delay in the communication and problem-solving domains of offspring, whereas no association was found between maternal pre-pregnancy underweight and offspring neurobehavioral development. These results were consistent with previous research findings. Neggers et al. (23) first reported a negative association between maternal pre-pregnancy obesity and childhood cognitive development. Using the differential ability scales (DAS), the study found that children born to mothers with pre-pregnancy obesity had significantly lower general cognitive ability (*β* = −4.7, *p* = 0.001) and nonverbal ability (*β* = −5.6, *p* = 0.003) compared to those born to mothers with normal pre-pregnancy weight. However, the study population was restricted to low-income African American women who also exhibited suboptimal plasma zinc levels. Huang et al. (24), using data from the U. S. Collaborative Perinatal Project (a large prospective cohort study including 30,212 women) found that children exposed to maternal pre-pregnancy obesity exhibited impaired full-scale IQ and verbal IQ scores. A cohort study from Australia further indicated that offspring of women with long-term pre-pregnancy obesity were at increased risk of developmental delays in gross and fine motor skills (RR = 1.64, 95% CI: 1.04–2.61), communication skills, and general cognition (RR = 1.71, 95% CI: 1.09–2.68). Maternal pre-pregnancy overweight/obesity has been associated with an increased risk of developmental delays in the communication and problem-solving domains in offspring, as supported by both this study and previous evidence, underscoring the clinical importance of implementing evidence-based targeted early interventions for this high-risk population. To mitigate communication delays, daily parent–child conversations, shared picture book reading, and responsive caregiving to infant vocalizations have been shown to effectively promote language development (25, 26). For problem-solving abilities, activities such as block building, puzzle games, and exploratory play are demonstrated to significantly enhance cognitive and executive functions (27). Furthermore, intervention programs such as the U. S. Early Head Start initiative, which has proven effective in high-risk pediatric populations, can serve as a valuable reference for designing comparable intervention strategies (28). Preliminary evidence suggested that maternal pre-pregnancy obesity may lead to chronic systemic inflammation and placental inflammatory responses, resulting in elevated levels of pro-inflammatory cytokines such as interleukin-6 (IL-6), interleukin-8 (IL-8), tumor necrosis factor-alpha (TNF-*α*), and C-reactive protein (CRP) (29, 30). Early-life exposure to an inflammatory environment can affect the development and function of fetal microglia, which are closely involved in key neurodevelopmental processes such as neuronal proliferation and differentiation, synaptogenesis, myelination, and the establishment of neural connectivity (31). Population-based studies further support this hypothesis, as demonstrated by the Viva project involving 1,361 mother–child pairs, where maternal inflammation was found to partially mediate the association between maternal obesity and offspring cognitive function (32). Studies on the prediction and prevention of preeclampsia and intrauterine growth restriction (IUGR) have shown that persistently elevated maternal inflammation during pregnancy increases the risk of neurodevelopmental delays in offspring across cognitive, motor, and social domains (11). Children born to mothers with the highest levels of inflammation exhibit the greatest number of affected neurodevelopmental areas. Other potential biological mechanisms include prenatal micronutrient deficiencies, alterations induced by metabolic hormones, and various maternal physiological changes associated with inappropriate gestational weight (33, 34). This study provides an important foundation for the development of early-life health intervention strategies and carries clear public health implications. First, it offers concrete and compelling scientific evidence for health literacy education targeting women of reproductive age. Whereas traditional preconception counseling often emphasizes “balanced diet,” our findings underscore the critical importance of controlling pre-pregnancy weight and reducing chronic inflammation as core messages. Healthcare providers can explicitly inform women that maintaining pre-pregnancy BMI within the healthy range (18.5–24.9 kg/m^2^) is not only relevant for aesthetics or metabolic health, but also essential for providing a low-inflammation intrauterine environment that supports optimal brain development in offspring. This reframes weight management as a new and crucial priority, which may enhance adherence and ultimately optimize perinatal weight control. Second, this study offers key scientific evidence for developing targeted maternal–child health interventions, highlighting that the intervention window should be extended to the pre-pregnancy and even preconception stage, rather than being limited to pregnancy. This perspective provides theoretical support for reforming current perinatal healthcare models. Collectively, our findings lay a solid scientific foundation for the design of tailored educational content, screening tools, and clinical intervention strategies, ultimately serving the overarching goal of improving long-term health outcomes in offspring.

Evidence suggested that early-life exposure to adverse environments may have sex-specific effects on child neurodevelopment (35). Consistent with these observations, our sex-stratified subgroup analyses revealed that maternal pre-pregnancy overweight/obesity exerted statistically significant detrimental effects on problem-solving and personal-social developmental domains, exclusively in male offspring. Previous research on child neurodevelopment has also indicated that boys are more susceptible to neurobehavioral developmental disorders, such as autism spectrum disorder (ASD), attention-deficit/hyperactivity disorder (ADHD), and schizophrenia. Several studies have reported that male preterm infants (gestational age <37 weeks) are at higher risk of neurodevelopmental impairments (36–38). However, when male infants exhibit poorer developmental outcomes following perinatal risk factors such as prematurity, it remains difficult to disentangle causal relationships. Similar to the findings of this study, previous reports have shown that boys appear to be more vulnerable than girls to the adverse effects of maternal pre-pregnancy overweight/obesity (39). Pre-pregnancy overweight/obesity were associated with lower IQ scores in 7-year-old boys, but not in girls. One possible explanation suggested by the research is the sex-specific differences in intrauterine growth rates: males and females develop at different rates in utero, and faster-growing fetuses may be more susceptible to prenatal insults (40). Other mechanisms may involve placental pathways (41). In light of the elevated neurodevelopmental risk observed in male offspring of mothers with pre-pregnancy overweight/obesity, sex-specific interventions are warranted. Prenatal strategies include tighter gestational weight gain control (7–9 kg for male-bearing overweight/obese mothers) and targeted nutrient supplementation—such as docosahexaenoic acid (DHA, ≥300 mg/day) and omega-3 fatty acids (1.2 g/day with EPA ≥ 500 mg and DHA ≥ 300 mg)—to mitigate neuroinflammation and support fetal brain development (42–44). Postnatal interventions should focus on enhancing problem-solving abilities through block play and hide-and-seek tasks, and improving social–emotional functioning via caregiver-infant interaction games and structured peer activities (45, 46). Long-term monitoring using sex-specific developmental benchmarks is recommended to enable early detection and timely intervention. Therefore, sex differences must be considered not only when analyzing the detrimental effects of prenatal exposures on neurodevelopment, but also when designing targeted early-life interventions for at-risk populations.

This prospective cohort study explored the causal relationship between pre-pregnancy BMI and repeated assessments of offspring neurobehavioral development from 1 to 36 months of age, with thorough adjustment for confounding factors, thereby enhancing the reliability of the findings. However, certain limitations remain. Given the complexity of child neurodevelopment, factors such as maternal diet and stress during pregnancy, postnatal environmental influences, and genetic factors were not included in the analysis. In addition, the assessment of offspring neurobehavioral development was based on parent-reported questionnaires, which may be subject to subjective bias. Future studies should incorporate additional factors such as maternal nutrition during pregnancy, psychological status, inflammatory profiles, and genetic background to conduct multidimensional analyses. At the same time, the inclusion of objective assessment tools (e.g., neuropsychological scales or neuroimaging measures) and extended follow-up into school age is recommended to more comprehensively elucidate the long-term impact and potential mechanisms of pre-pregnancy BMI on offspring neurodevelopment. Such approaches would provide more precise scientific evidence to guide clinical practice and public health interventions.

## Data Availability

The original contributions presented in the study are included in the article/supplementary material, further inquiries can be directed to the corresponding authors.
